# Rheumatology education in US pediatric residency programs: results of a comprehensive program director survey

**DOI:** 10.1186/1546-0096-10-S1-A7

**Published:** 2012-07-13

**Authors:** Megan L Curran, Yasmin Husain

**Affiliations:** 1 Children's Memorial Hospital, Chicago, IL, USA; 2 University of Pennsylvania Medical School, Philadelphia, PA, USA

## Purpose

Rheumatology is one of the smallest subspecialties in the field of pediatrics. Due to the deficiency of pediatric rheumatologists in the U.S., there is a shortage of educators. Previous research showed that 40% of pediatric residency programs have no pediatric rheumatologist on faculty. Although previous studies described the availability of rheumatology training in pediatric residency programs, little is known about the educational content or methods. To develop useful educational curricula for residency program use, rheumatology educators need to understand how pediatric residents are currently taught about rheumatology as well as residency programs’ perceived educational needs and barriers.

## Methods

In the summer of 2010, all pediatric residency program directors in the U.S. and U.S. territories were sent email and paper surveys. Contact information was collected using databases from the American College of Graduate Medical Education and the American Medical Association. Email surveys were sent twice via Survey Monkey three weeks apart. One paper survey and a reminder postcard were sent via U.S. mail.

## Results

115 of 197 (58.4%) program directors have responded to date. 68% of programs were university based, 26% community based and 5% were described as “other.” 65% said that their institution employed pediatric rheumatologists, 34% had no rheumatologist and 1% were unsure. 69% of responding programs lack a required rheumatology rotation. 29% have required rotations supervised by rheumatologists: 12% include both inpatient and outpatient experience, 8% only inpatient and 9% only outpatient. 1% of programs have a required rotation not supervised by a rheumatologist. Educational goals and objectives are not provided to residents in 26% of programs. Of 91 responding programs, rheumatology material is taught by general pediatricians in 73%, orthopedists in 56%, faculty rheumatologists in 53%, visiting pediatric rheumatologists in 23%, chief residents in 23% and senior residents in 15%. Rheumatology is taught via inpatient rounds in 78/87 programs (90%), lectures with slides in 83/88 (94%), informal lecture in 54/77 (70%), demonstration of musculoskeletal exam in 78/87 (90%), video in 6/64 (9%), case based discussion in 62/76 (82%), online coursework in 4/63 (6%), and by board question review in 77/83 (93%). More program directors indicated that residents like live musculoskeletal exam demonstration (95%) compared to video demonstration (59%). Interactive case based lectures and Jeopardy games were perceived to be well-liked at 98% and 90%. Many programs would spend more time teaching rheumatology if a curriculum was provided. Teaching by general pediatricians, chief residents and senior residents would increase. 55% agreed that having insufficient faculty is a barrier to rheumatology education. 42% agreed that poor access to teaching resources is a barrier.

## Conclusion

Our survey results provide a detailed picture of how rheumatology is taught in pediatric residency and how teaching can be improved. This information is a foundation for the development of teaching materials that will be targeted for residency programs with little rheumatologist contact.

## Disclosure

Megan L. Curran: None; Yasmin Husain: None.

